# Efficacy and safety of tofacitinib on COVID-19 patients: A systematic review and meta-analysis

**DOI:** 10.1016/j.heliyon.2024.e38229

**Published:** 2024-09-20

**Authors:** Gofarana Wilar, Cecep Suhandi, Kohji Fukunaga, Ichiro Kawahata

**Affiliations:** a Department of Pharmacology and Clinical Pharmacy, Faculty of Pharmacy, Universitas Padjadjaran, Sumedang, 45363, Indonesia; b Department of Pharmaceutics and Pharmaceutical Technology, Faculty of Pharmacy, Universitas Padjadjaran, Sumedang, 45363, Indonesia; c Department of Pharmacology, Graduate School of Pharmaceutical Sciences, Tohoku University, Sendai, 980-8578, Japan; d Department of CNS Drug Innovation, Graduate School of Pharmaceutical Sciences, Tohoku University, Sendai, 980-8578, Japan

**Keywords:** Tofacitinib, Janus kinase inhibitor, Coronavirus disease 2019, SARS-CoV-2, Cytokine syndrome

## Abstract

The use of drugs off-label for managing COVID-19 offers a potential approach. Among these potential drugs, tofacitinib, a JAK inhibitor, is strongly implicated in its ability to mitigate mortality by attenuating the cytokine storm syndrome. This study systematically reviewed and quantitatively assessed the effectiveness and safety profile of tofacitinib use through meta-analysis. Through searches of the PubMed, Scopus, and the Cochrane Library databases up to May 31, 2024, six articles meeting inclusion criteria were identified, encompassing 669 patients diagnosed with COVID-19. The review findings indicate that tofacitinib use demonstrates significant clinical efficacy, as evidenced by a reduced risk of mortality (P = 0.003), and a decreased need for invasive mechanical ventilation (P = 0.0002). Furthermore, tofacitinib use is not correlated with an increased risk of adverse drug reactions (P = 0.98), indicating a favorable safety profile. In conclusion, the evidence supports the clinical efficacy of tofacitinib for COVID-19 patients without concomitant risks of adverse effects. Further clinical studies, especially larger-scale randomized controlled trials, are necessary to validate the findings of this study.

## Highlights

• Tofacitinib, a JAK inhibitor, shows promise in mitigating COVID-19 mortality by reducing cytokine storm syndrome.
• Meta-Analysis of six studies with six hundred sixty-nine COVID-19 patients indicates significant clinical efficacy.
• Tofacitinib use reduces mortality risk and need for invasive mechanical ventilation.
• No increased risk of adverse drug reactions, suggesting a favorable safety profile.

## Graphical abstract

**Figure:**
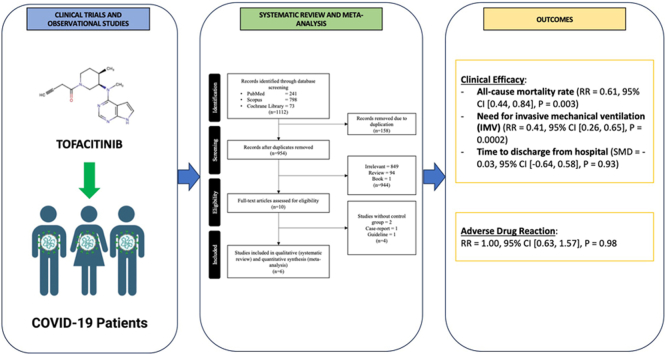

## 1 Introduction

Although the cases of Coronavirus Disease 2019 (COVID-19) infections have declined, it does not imply the complete eradication of severe acute respiratory syndrome coronavirus 2 (SARS-CoV-2) infections. Every day, there are still patients infected with COVID-19 requiring treatment [1]. The emergence of novel virus variants poses an additional challenge, potentially compromising vaccine efficacy [2]. Generally, COVID-19 patients undergo three primary infection phases [3]. Beginning with an initial phase characterized by mild symptoms, the infection progresses to a pulmonary phase marked by heightened immune system activation due to increased viral load [4]. In the final phase, known as the hyperinflammatory phase, patients experience excessive inflammation leading to tissue damage and respiratory failure as a leading cause of death [5]. The hyperinflammatory condition is instigated by a phenomenon termed the "cytokine storm," where the body secretes cytokines (interleukin-6 (IL-6) and tumor necrosis factor α (TNF‐α)) in large quantities in response to the high immunogenicity of SARS-CoV-2 [6,7]. Cytokine storm has been identified as a primary cause of death in COVID-19 infections, with a mortality prevalence reaching 23.1 % [8].

Developing effective treatments for cytokine storm is crucial for improving outcomes and reducing mortality rates in severe COVID-19 cases. In an effort to address these challenges, previously available specific agents inhibiting IL-6 or TNF-α have been assessed for their efficacy in combating cytokine storm in COVID-19 infections [9,10]. However, a randomized controlled trial (RCT) indicated that the use of tocilizumab as an anti-IL-6 agent was ineffective in preventing deaths attributed to cytokine storm in hospitalized patients with moderate-level severity [11]. Furthermore, a meta-analysis of randomized controlled trials (RCTs) investigating tocilizumab revealed that its use was not associated with a reduced risk of death; instead, it increased the risk of secondary infections [12]. Moreover, an RCT also demonstrated that the administration of infliximab, a TNF-α inhibitor, did not significantly improve the recovery time of hospitalized patients with COVID-19 infection [13]. These findings emphasize the ongoing need for alternative potential agents capable of addressing the detrimental progression resulting from cytokine storm.

The exploration of off-label utilization of alternative immunomodulatory agents with unique mechanisms but relevance to cytokine storm emerges as a swift strategy in addressing this issue [14]. Notably, intriguing insights have emerged regarding baricitinib, a Janus kinase (JAK) inhibitor, which has demonstrated effectiveness in fostering clinical improvements among hospitalized COVID-19 patients [15]. Both the RECOVERY and BARRIER randomized controlled trials (RCTs) consistently reported the efficacy of baricitinib in reducing mortality rates [16,17]. These findings are corroborated by a meta-analysis of RCTs, suggesting a decline in 28-day mortality rates [18]. Nevertheless, its utility hasn't fully translated to mitigating disease progression, such as reducing the requirement for invasive mechanical ventilation (IMV). Despite this, the findings on baricitinib suggest a promising avenue, demonstrating the effectiveness of JAK inhibitors in addressing the cytokine storm caused by the coronavirus. This opens avenues for exploring other JAK inhibitors like tofacitinib, which may potentially outperform baricitinib [19]. Evidence supporting this notion comes from a retrospective study demonstrating tofacitinib's ability to lower mortality rates in COVID-19 patients with C-reactive protein (CRP) levels ranging from 60 to 150 mg/dL [20]. To substantiate this hypothesis, this systematic review and meta-analysis were performed to assess the effectiveness and safety of tofacitinib in managing COVID-19 patients, incorporating observational studies to augment findings due to limited RCTs.

## 2 Materials and methods

This research strictly adheres to established protocols, emphasizing the guidelines outlined in the Preferred Reporting Items for Systematic Reviews and Meta-Analyses (PRISMA) [21]. Additionally, several methodological studies related to meta-analysis were referenced to enhance the accuracy and reliability of the analytical methods [[22], [23], [24]]. The importance of adhering to a protocol in this study is highlighted by our registration with the International Prospective Register of Systematic Reviews (PROSPERO), where we are assigned the registration code CRD42024531118, offering a transparent framework for the execution of this investigation.

### 2.1 Literature search

A thorough search of the PubMed, Scopus, and Cochrane Library databases was performed to locate relevant publications released between March 1, 2020, and May 31, 2024. The search employed these specific keywords: (COVID-19 OR "Coronavirus Disease" OR SARS-CoV-2) AND (Tofacitinib OR "JAK Inhibitor" OR "Janus Kinase Inhibitor"). All retrieved results were imported into Microsoft Excel 2016, where identical findings were identified and removed. The search outcomes were restricted solely to clinical studies, including both RCTs and non-RCTs, as well as observational studies involving human subjects and published in English. Articles such as reviews, letters, case reports, opinion articles, abstracts, and short reports were excluded.

### 2.2 Inclusion criteria and study selection

The inclusion criteria comprised: (1) Patients: adult patients with confirmed diagnosis of COVID-19 through laboratory testing; (2) Intervention: the intervention group included patients receiving treatment with tofacitinib outside standard management, compared to a control group receiving either a placebo or standard-of-care treatments as baseline; (3) Control: the control group encompassed patients receiving either a placebo or standard-of-care treatments; (4) Outcomes: clinical efficacy (all-cause mortality, need for invasive mechanical ventilation (IMV), and time to discharge from hospital) and adverse drug reactions (e.g., urinary tract infections, upper respiratory tract infections, diarrhea, headache, arthralgia, and thromboembolism).

In the initial phase of selecting studies, two reviewers independently assessed articles by reviewing their titles and abstracts according to the predetermined inclusion criteria. Subsequently, full-text articles were obtained to undergo further analysis regarding the eligibility of included studies. Any disparities were addressed either through dialogue between the reviewers or by involving a third reviewer for further input.

### 2.3 Data extraction and quality assessment

The data extraction and quality assessment were independently performed by two reviewers. Pertinent information, including author details, study types, locations, number of participants, follow-up durations, dosage of tofacitinib used, and outcomes, was tabulated. The methodological quality of randomized controlled trials (RCTs) was assessed using the Risk of Bias (RoB) assessment tool from the Cochrane Handbook, while the quality of non-RCT clinical studies and observational studies was evaluated using the Newcastle-Ottawa Scale (NOS) [25,26]. Each RCT was evaluated for low risk, high risk, or unclear risk across various criteria including sequence generation, concealment of allocation, blinding of outcome assessors, incomplete outcome data, selective reporting of outcomes, and other potential biases [25]. The NOS assessed the quality of non-RCT clinical studies and observational studies through eight questions grouped into three categories: selection of participants, group comparability, and outcome evaluation. The maximum score was 9, with higher scores indicating better study quality [26]. Any discrepancies between the reviewers were resolved through discussion or consultation with a third reviewer.

### 2.4 Statistical analysis

RevMan 5.4 was utilized to conduct the statistical analysis. Risk ratios (RR) were applied to dichotomous outcomes, while standardized mean differences (SMD) were used for continuous variables. Both measures of effect were reported along with 95 % confidence intervals (CI). Given the variability in participant numbers across trials, all results were computed using a random effects model [27]. Statistical heterogeneity for each pairwise comparison was evaluated using the I^2^ statistic, with values classified as low (<25 %), moderate (25–50 %), and high (>50 %) heterogeneity [27]. Subgroup and sensitivity analyses were conducted to investigate sources of heterogeneity when sufficient data were available. Egger's test and funnel plots were employed to identify potential publication bias when more than ten trials were included for an outcome [27,28]. Statistical significance was defined as a P-value of less than 0.05.

## 3 Results

### 3.1 Search results

**Fig. 1:**
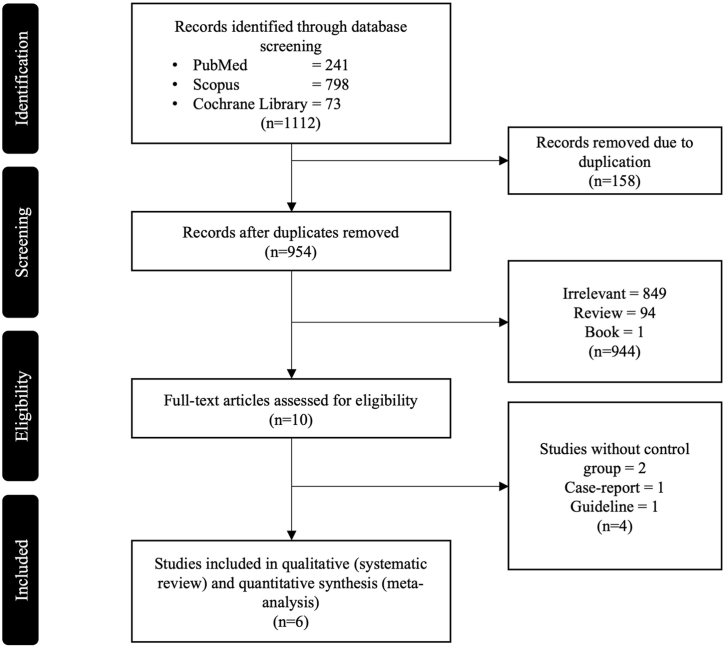
Flowchart of literature search.

The literature search across all databases yielded a total of 1112 articles. Among these, 158 were identified as duplicates and subsequently removed. Following a meticulous review of titles and abstracts, 944 articles were deemed ineligible based on the inclusion criteria. From the remaining pool of 10 articles, a full-text screening process revealed that two literatures did not incorporate control groups, one literature was a case report, and another article was a guideline document. Consequently, only 6 articles remained that met the eligibility criteria and were deemed suitable for further qualitative and quantitative analysis [20,[29], [30], [31], [32], [33]]. The literature selection process is depicted in Fig. 1.

### 3.2 Study characteristics

Detailed information regarding the characteristics of the included studies is presented in Table 1. Among them, three are randomized controlled trials (RCTs) [30,31,33], one is a non-RCT clinical trial [32], and the remaining two are observational studies in the form of retrospective studies [20,29]. The studies were conducted across diverse locations, including Iran, Italy, Brazil, Russia, and India. In total, 669 confirmed COVID-19 patients were involved across all included studies, with 338 patients receiving tofacitinib and 331 serving as the control group. The administered doses of tofacitinib varied, ranging from 5 mg twice daily to 10 mg twice daily and 20 mg daily. The follow-up period for study subjects varied from fourteen to fifty days. The consistency of outcomes across all included studies encompassed mortality rate, the need for invasive mechanical ventilation (IMV), length of hospital stay, and incidence of adverse drug reactions.

**Table 1:** Characteristics of included studies.

Author and year	Study types	Study location	Number of participants		Follow-up durations (days)	Dosage of tofacitinib used	Outcomes
			Tofacitinib	Control			
Almasi et al. (2023) [32]	Non-RCT clinical trial	Iran	29	23	14	5 mg twice daily	The primary outcomes: heart rate, breath count, radiographic alterations, oxygen saturation, and the consciousness level. Secondary outcomes: serum levels of inflammatory cytokines (TNF-α, IL-2, IL-6), along with blood biomarkers (D-dimer, ferritin, lactate dehydrogenase (LDH), white blood cell count, hemoglobin, and platelet count.
Ferrarini et al. (2023) [31]	Randomized controlled trial (RCT)	Italia	58	58	28	10 mg twice a day	The primary outcomes: percentage of patients requiring non-invasive or invasive mechanical ventilation. Secondary outcomes: percentage of patients showing clinical improvement by day 7, percentage of patients requiring admission to the ICU, and mortality rate.
Guimarães et al. (2021) [33]	Randomized controlled trial (RCT)	Brazil	144	145	28	10 mg twice daily	The primary outcome: mortality rate or respiratory failure. Secondary outcomes: status of being alive without mechanical ventilation, status of being alive without hospitalization, cure rate, duration of hospital stay, duration of ICU stay, and occurrence and severity of adverse events.
Maslennikov et al. (2021) [20]	Retrospective study	Russia	32	30	50	10 mg twice a day on the first day, and 5 mg twice a day for 4 days	The primary outcome: survival rate. Secondary outcomes: the length of hospital stay, frequency of ICU admission and mechanical ventilation, overall disease duration, alterations in key biomarker levels, respiratory function 7–10 days following the initiation of tofacitinib treatment, and chest CT findings.
Murugesan et al. (2021) [30]	Randomized controlled trial (RCT)	India	50	50	28	10 mg twice a day	The primary outcome: the percentage of patients who did not need mechanical ventilation, high-flow oxygen, or ECMO by day 7, or who did not experience mortality. Secondary outcomes: the variations in inflammatory marker levels, such as serum ferritin, interleukin-6 (IL6), and CRP from baseline to day 7, and alterations in clinical and radiological manifestations.
Singh et al. (2021) [29]	Retrospective case-control study	India	25	25	21	20 mg daily	The primary outcome: the World Health Organization (WHO) ordinal scale in their R & D blueprint document. Secondary outcomes: levels of inflammatory biomarkers, viral clearance, and treatment-related adverse effects.

**Fig. 2:**
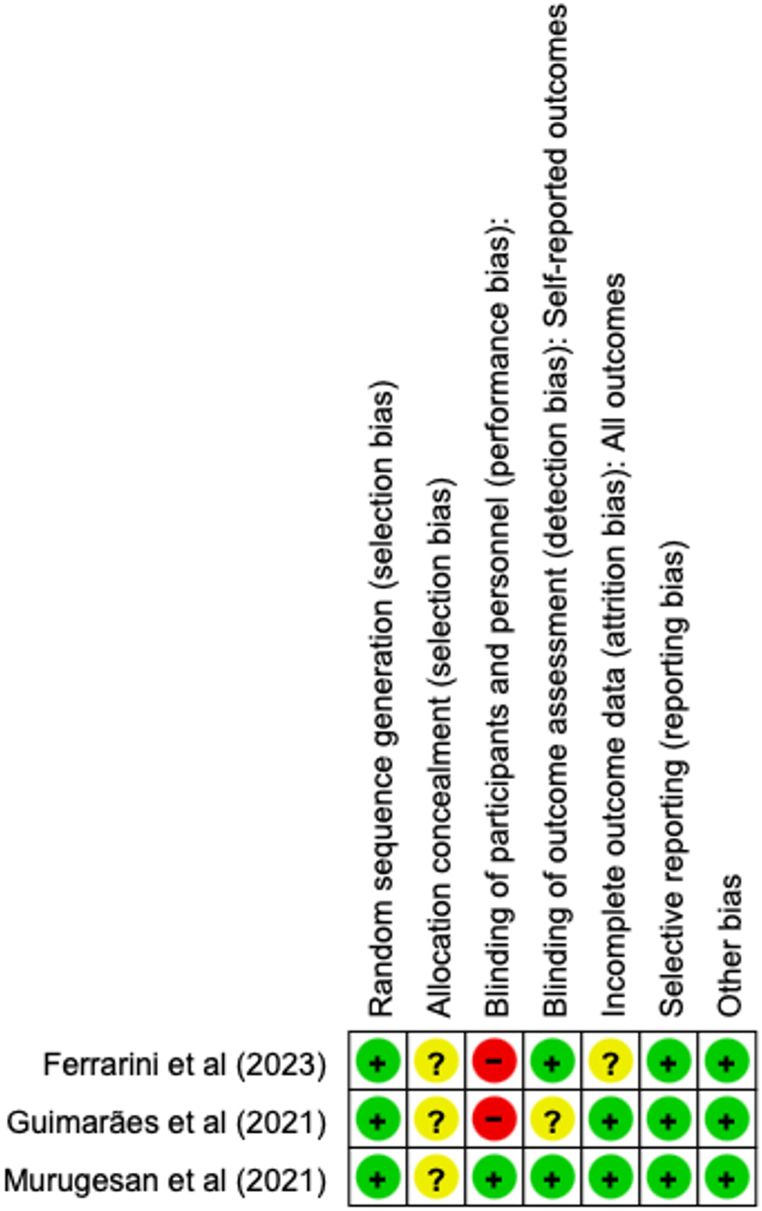
Quality assessment of randomized controlled trials (RCTs).

The quality of the included randomized controlled trials (RCTs) is depicted in Fig. 2. All included RCTs exhibited low risk in selection bias, reporting bias, and other biases. However, two out of the three RCTs [30,31] showed a high risk of performance bias due to the open-label nature of the studies, wherein study subjects were aware of the treatment they would receive. Overall, the three encompassed RCTs demonstrated a moderate to high risk of bias.

The quality of three studies (one non-RCT clinical trial and two retrospective studies) [20,29,32] was evaluated employing the Newcastle-Ottawa Scale (NOS) (Table 2). All studies provided information on case definition and definition and comparability of controls adequately. However, only one study provided adequate information regarding the selection of controls [20]. Information regarding exposure was well-described across all studies, although none of them included information on the non-response rate. Overall, the three studies obtained NOS scores with an average of 7.3. In terms of quality, these three studies demonstrated moderate to high quality.

**Table 2:** Quality assessment of non-RCT clinical trials and observational studies.

Study	Is the Case Definition Adequate?	Representativeness of the Cases	Selection of Controls	Definition of Controls	Comparability of Cases and Controls	Ascertainment of Exposure	Use the same method to determine case and control exposure factors	Non-Response Rate	Total
Almasi et al. (2023) [32]	∗	∗		∗	∗∗	∗	∗		7
Maslennikov et al. (2021) [20]	∗	∗	∗	∗	∗∗	∗	∗		8
Singh et al. (2021) [29]	∗	∗		∗	∗∗	∗	∗		7

### 3.3 Clinical efficacy

#### 3.3.1 All-cause mortality rate

**Fig. 3:**
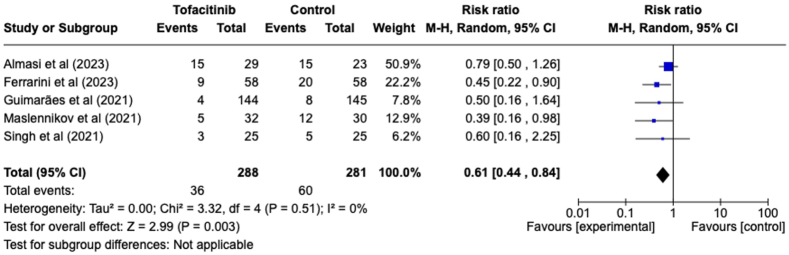
Forest plot of all-cause mortality outcome between tofacitinib and control group.

Five studies evaluated the efficacy of tofacitinib in reducing mortality rates (Fig. 3). The group receiving tofacitinib exhibited a tendency towards lower mortality (RR = 0.61, 95 % CI [0.44, 0.84]). The decrease in the risk of mortality with tofacitinib was statistically significant (P = 0.003). There was no significant heterogeneity detected among the five evaluated studies (I^2^ = 0 %).

#### 3.3.2 Need for invasive mechanical ventilation (IMV)

**Fig. 4:**
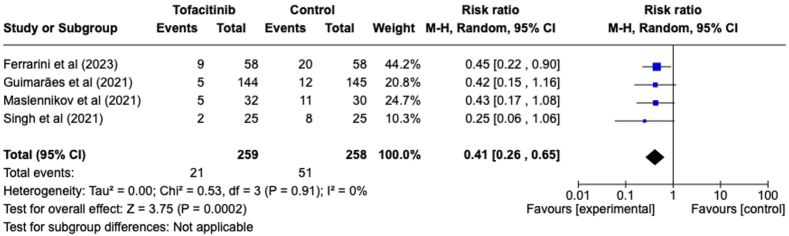
Forest plot of patients need for IMV outcome between tofacitinib and control group.

Four studies assessed the need for patients to undergo invasive mechanical ventilation (IMV) (Fig. 4). There was a significant decrease in the need for IMV among patients compared to the control group (RR = 0.41, 95 % CI [0.26, 0.65], P = 0.0002). Evaluation of these four studies did not demonstrate heterogeneity (I^2^ = 0 %).

#### 3.3.3 Time to discharge from hospital

**Fig. 5:**
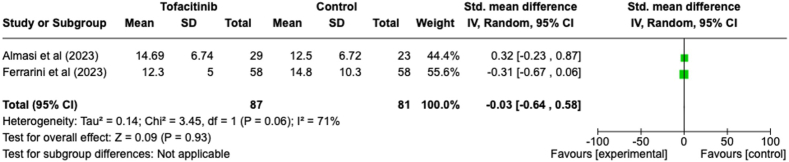
Forest plot of time to discharge from hospital outcome between tofacitinib and control group.

Only two studies evaluated the time required for discharge from the hospital (Fig. 5). The evaluation of these two studies did not yield positive findings; instead, it indicated an increase in the time needed for discharge from the hospital (SMD = −0.03, 95 % CI [−0.64, 0.58]). However, the results obtained were not statistically significant (P = 0.93). Additionally, there was a high level of heterogeneity detected in the evaluation of this parameter (I^2^ = 71 %).

### 3.4 Adverse drug reactions

**Fig. 6:**
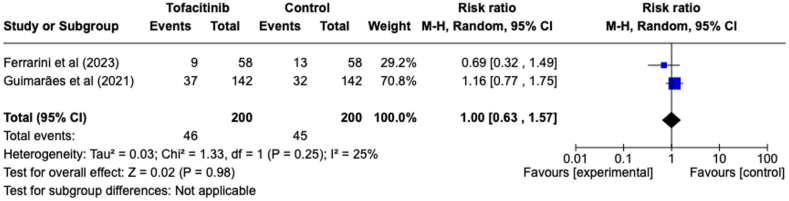
Forest plot of adverse drug reactions outcome between tofacitinib and control group.

Only two studies provided an evaluation of the likelihood of adverse effects arising from the use of tofacitinib (Fig. 6). There was no statistically significant difference observed in the incidence of adverse reactions between the tofacitinib and control groups (RR = 1.00, 95 % CI [0.63, 1.57], P = 0.98). This finding indicates that the use of tofacitinib does not lead to an increase in adverse drug reactions. Although the included studies were limited, heterogeneity in this evaluation was low to moderate (I^2^ = 25 %).

## 4 Discussion

This review comprised six articles meeting the inclusion criteria, collectively involving 669 COVID-19 patients. The findings indicated that the utilization of tofacitinib among COVID-19 patients was associated with decreased all-cause mortality and a reduced requirement for invasive mechanical ventilation (IMV) from a clinical efficacy standpoint. Furthermore, its administration exhibited a favorable safety profile, as demonstrated by the lack of significant differences in the incidence of adverse drug reactions between the tofacitinib-receiving group and the control group. These findings highlight the potential clinical implications derived from this systematic review and meta-analysis.

**Fig. 7:**
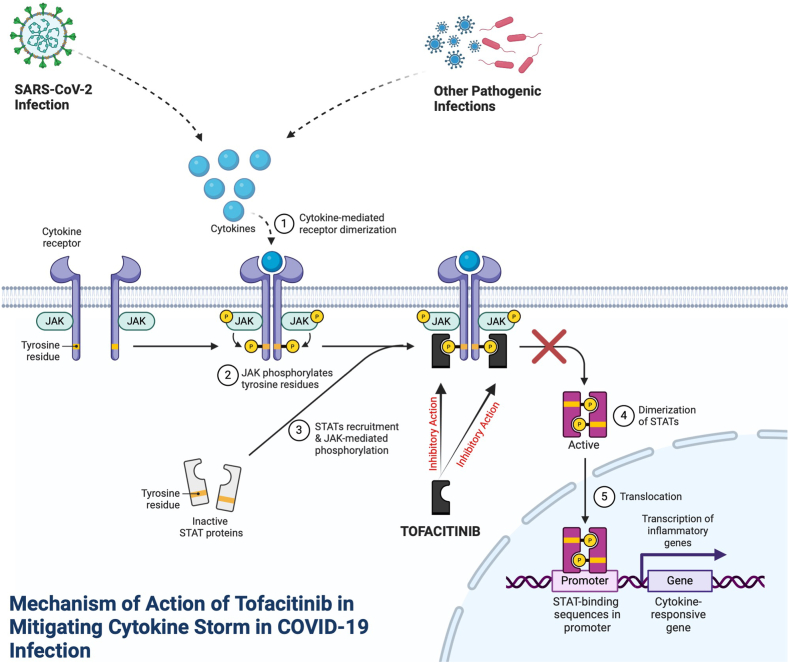
Mechanism of action of tofacitinib in addressing cytokine storm in COVID-19 infection.

Various clinical evidence suggests that patients with COVID-19 experience poor progression due to hyperinflammatory conditions. The high immunogenicity of SARS-CoV-2 draws attention to the immune system's release of various cytokines, leading to the onset of "cytokine storm" conditions [14]. Excessive cytokine release, including IL-6, TNF-α, IFNα, INFβ, and INFγ, induces inflammation in the respiratory tract, resulting in airway closure, respiratory failure, and ultimately death [34]. Several systematic reviews have demonstrated that the use of immunosuppressants can provide positive benefits in managing COVID-19 patients, particularly in preventing poor prognoses due to cytokine storm syndrome [35,36]. Tofacitinib, as one of the commonly used immunosuppressive agents for treating rheumatoid arthritis, is also expected to contribute to the acceleration of COVID-19 drug development. By targeting the JAK/STAT signaling pathway, tofacitinib can prevent further actions induced by various cytokines, including those released during SARS-CoV-2 attacks (Fig. 7) [37]. Building on the success of tofacitinib in managing cytokine storm in COVID-19 patients, it is highly likely that the benefits of tofacitinib will extend beyond treating COVID-19. Since cytokine storms can also occur in other respiratory infections, including those caused by the influenza virus, this possibility should be considered [38].

As with the use of immunosuppressants in general, the use of tofacitinib carries the risk of common side effects typically arising from decreased immune response to other pathogenic infections (Fig. 7). Tofacitinib is often associated with an increased risk of infections in the urinary tract and upper respiratory tract [39]. Additionally, a high risk of hyperlipidemia has been observed in patients receiving tofacitinib treatment [40]. Other reported side effects of tofacitinib use include diarrhea, headache, pain in extremities, and arthralgia [41]. Considering that tofacitinib is used in patients with conditions like COVID-19 that impair the immune system, evaluating the potential risk of side effects is crucial. In an open-label randomized controlled trial conducted by Ferrarini et al., the use of tofacitinib showed a lower risk of serious infection compared to the control group (3.5 % vs. 4.2 %) [31]. Furthermore, in another RCT conducted by Guimarães et al., not a single case of bacterial infection due to tofacitinib use was reported in COVID-19 patients [33]. Its use also did not result in thromboembolism, indicating that the use of tofacitinib at a dosage of 10 mg twice daily does not pose a risk of causing hyperlipidemia. These findings serve as important evidence supporting the results obtained in this review.

Based on our exploration, this systematic review and meta-analysis stand as the first to comprehensively address the clinical efficacy and safety of tofacitinib use in COVID-19 patients. In an effort to enhance the study's comprehensiveness, not only clinical trials, both randomized controlled trials (RCTs) and non-RCTs, were included, but also several observational studies. Specifically, there were four clinical studies included, comprising three RCTs and one non-RCT clinical study. Meanwhile, the other two included articles were observational studies in the form of retrospective studies.

Nevertheless, this review is not devoid of limitations. The primary limitation of this meta-analysis is the limited number of studies, encompassing only six studies with a total population size of 669 subjects. Secondly, the evaluation of several parameters such as the time needed for hospital discharge and adverse drug reactions indicates mild and high heterogeneity, respectively. This limitation may impact the heterogeneity of the statistical analyses conducted. Thirdly, given the emergence of potential heterogeneity, subgroup and sensitivity analyses could serve as solutions to identify the source of heterogeneity. Unfortunately, due to the insufficient number of included studies (less than 10), both analyses could not be performed. Lastly, by including studies with an open-label system, the potential for performance bias may arise due to the lack of blinding of participants and personnel.

## 5 Conclusion

Overall, this study indicates that the use of tofacitinib provides positive benefits for COVID-19 patients by reducing mortality rates and the need for invasive mechanical ventilation (IMV). Additionally, tofacitinib does not show a correlation with a higher risk of adverse drug reactions. However, the limited number of literature and the moderate quality of studies underscore the need for high-quality randomized controlled trials (RCTs) in the future to further validate these findings.

## Funding

This work is primarily supported by the Padjadjaran Recharging Academic Grant (GW) 2024. It is also supported by additional funding from the Indonesian Endowment Found (GW) and Kakenhi (KF).

## Consent for publication

Not applicable.

## Ethics approval and consent to participate

Ethical approval was not needed because this is a meta-analysis.

## Availability of data and material

Data will be made available on request.

## CRediT authorship contribution statement

**Gofarana Wilar:** Writing – review & editing, Validation, Project administration, Funding acquisition, Formal analysis, Conceptualization. **Cecep Suhandi:** Writing – original draft, Software, Methodology, Formal analysis, Conceptualization. **Kohji Fukunaga:** Writing – review & editing, Validation, Investigation. **Ichiro Kawahata:** Writing – review & editing, Validation, Investigation, Formal analysis.

## Declaration of competing interest

The authors declare that they have no known competing financial interests or personal relationships that could have appeared to influence the work reported in this paper.

## References

[1] Guo Y.R., Cao Q.D., Hong Z.S., Tan Y.Y., Chen S.D., Jin H.J., Sen Tan K., Wang D.Y., Yan Y. (2020). The origin, transmission and clinical therapies on coronavirus disease 2019 (COVID-19) outbreak- A n update on the status. Mil Med Res.

[2] Caldwell J.M., Le X., McIntosh L., Meehan M.T., Ogunlade S., Ragonnet R., O'Neill G.K., Trauer J.M., McBryde E.S. (2021). Vaccines and variants: Modelling insights into emerging issues in COVID-19 epidemiology. Paediatr. Respir. Rev..

[3] Turk C., Turk S., Malkan U.Y., Haznedaroglu I.C. (2020). Three critical clinicobiological phases of the human SARS-associated coronavirus infections. Eur. Rev. Med. Pharmacol. Sci..

[4] Aguilar R.B., Hardigan P., Mayi B., Sider D., Piotrkowski J., Mehta J.P., Dev J., Seijo Y., Camargo A.L., Andux L., Hagen K., Hernandez M.B. (2020). Current understanding of COVID-19 clinical course and investigational treatments. Front. Med..

[5] Mason R.J. (2020). Pathogenesis of COVID-19 from a cell biology perspective. Eur. Respir. J..

[6] Tanner T., Wahezi D.M. (2020). Hyperinflammation and the utility of immunomodulatory medications in children with COVID-19. Paediatr. Respir. Rev..

[7] Gustine J.N., Jones D. (2021). Immunopathology of hyperinflammation in COVID-19. Am. J. Pathol..

[8] Ramatillah D.L., Gan S.H., Pratiwy I., Sulaiman S.A.S., Jaber A.A.S., Jusnita N., Lukas S., Bakar U.A. (2022). Impact of cytokine storm on severity of COVID-19 disease in a private hospital in West Jakarta prior to vaccination. PLoS One.

[9] Majidpoor J., Mortezaee K. (2022). Interleukin-6 in SARS-CoV-2 induced disease: interactions and therapeutic applications. Biomed. Pharmacother..

[10] Guo Y., Hu K., Li Y., Lu C., Ling K., Cai C., Wang W., Ye D. (2022). Targeting TNF-α for COVID-19: recent advanced and controversies. Front. Public Health.

[11] Salama C., Han J., Yau L., Reiss W.G., Kramer B., Neidhart J.D., Criner G.J., Kaplan-Lewis E., Baden R., Pandit L., Cameron M.L., Garcia-Diaz J., Chávez V., Mekebeb-Reuter M., Lima de Menezes F., Shah R., González-Lara M.F., Assman B., Freedman J., Mohan S.V. (2021). Tocilizumab in patients hospitalized with covid-19 pneumonia. N. Engl. J. Med..

[12] Boppana T.K., Mittal S., Madan K., Mohan A., Hadda V., Guleria R. (2022). Tocilizumab for COVID-19: a systematic review and meta-analysis of randomized controlled trials. Monaldi Arch. Chest Dis..

[13] O'Halloran J.A., Ko E.R., Anstrom K.J. (2023). Abatacept, cenicriviroc, or infliximab for treatment of adults hospitalized with COVID-19 pneumonia: a randomized clinical trial. JAMA.

[14] Rangappa P. (2021). Cytokine storm and immunomodulation in covid-19. Indian J. Crit. Care Med..

[15] Zhang X., Zhang Y., Qiao W., Zhang J., Qi Z. (2020). Baricitinib, a drug with potential effect to prevent SARS-COV-2 from entering target cells and control cytokine storm induced by COVID-19. Int. Immunopharm..

[16] Abani O., Abbas A., Abbas F. (2022). Baricitinib in patients admitted to hospital with COVID-19 (RECOVERY): a randomised, controlled, open-label, platform trial and updated meta-analysis. Lancet.

[17] Marconi V.C., Ramanan A.V., de Bono S. (2021). Efficacy and safety of baricitinib for the treatment of hospitalised adults with COVID-19 (COV-BARRIER): a randomised, double-blind, parallel-group, placebo-controlled phase 3 trial. Lancet Respir. Med..

[18] Selvaraj V., Finn A., Lal A., Khan M.S., Dapaah-Afriyie K., Carino G.P. (2022). Baricitinib in hospitalised patients with COVID-19: a meta-analysis of randomised controlled trials. EClinicalMedicine.

[19] Chebbi P., Shobha V., Rao V.K., Haridas V., Janardana R., Pinto B., Kumar S., Patil A., Tekkatte R., Salanke M., Mahendranath K.M. (2023). Occurrence and outcome of COVID-19 in AIRD patients on concomitant treatment with Tofacitinib- results from KRA COVID COHORT (KRACC) subset. BMC Rheumatol.

[20] Maslennikov R., Ivashkin V., Vasilieva E., Chipurik M., Semikova P., Semenets V., Russkova T., Levshina A., Grigoriadis D., Magomedov S., Efremova I., Dzhakhaya N. (2021). Tofacitinib reduces mortality in coronavirus disease 2019 Tofacitinib in COVID-19. Pulm. Pharmacol. Ther..

[21] Moher D., Liberati A., Tetzlaff J., Altman D.G., Antes G., Atkins D., Barbour V., Barrowman N., Berlin J.A., Clark J., Clarke M., Cook D., D'Amico R., Deeks J.J., Devereaux P.J., Dickersin K., Egger M., Ernst E., Gøtzsche P.C., Grimshaw J., Guyatt G., Higgins J., Ioannidis J.P.A., Kleijnen J., Lang T., Magrini N., McNamee D., Moja L., Mulrow C., Napoli M., Oxman A., Pham B., Rennie D., Sampson M., Schulz K.F., Shekelle P.G., Tovey D., Tugwell P. (2009). Preferred reporting items for systematic reviews and meta-analyses: the PRISMA statement. PLoS Med..

[22] Tian J., Zhang J., Ge L., Yang K., Song F. (2017). The methodological and reporting quality of systematic reviews from China and the USA are similar. J. Clin. Epidemiol..

[23] Li L., Tian J., Tian H., Moher D., Liang F., Jiang T., Yao L., Yang K. (2014). Network meta-analyses could be improved by searching more sources and by involving a librarian. J. Clin. Epidemiol..

[24] Xiu-Xia L., Ya Z., Yao-Long C., Ke-Hu Y., Zong-Jiu Z. (2015). The reporting characteristics and methodological quality of Cochrane reviews about health policy research. Health Pol..

[25] Higgins J.P.T., Altman D.G., Gøtzsche P.C., Jüni P., Moher D., Oxman A.D., Savović J., Schulz K.F., Weeks L., Sterne J.A.C. (2011). The Cochrane Collaboration's tool for assessing risk of bias in randomised trials. BMJ (Online).

[26] Wells G., Shea B., O'Connell D., Peterson J., Welch V., Losos M., Tugwell P. (2004). He Newcastle–Ottawa Scale (NOQAS) for Assessing the Quality of Non-randomized Studies in Meta-Analysis, The Ottawa Hospital.

[27] Higgins J.P.T., Thompson S.G., Deeks J.J., Altman D.G. (2003). Measuring inconsistency in meta-analyses. Br. Med. J..

[28] Lau J., Ioannidis J.P.A., Terrin N., Schmid C.H., Olkin I. (2006). The case of the misleading funnel plot. Br. Med. J..

[29] Singh P.K., Lalwani L.K., Govindagoudar M.B., Aggarwal R., Chaudhry D., Kumar P., Gehlaut P. (2021). Tofacitinib associated with reduced intubation rates in the management of severe covid-19 pneumonia: a preliminary experience. Indian J. Crit. Care Med..

[30] Murugesan H., Cs G., Nasreen H.S., Santhanam S., M G., Ravi S., Es S.S. (2022). An evaluation of efficacy and safety of tofacitinib, A JAK inhibitor in the management of hospitalized patients with mild to moderate COVID-19 - an open-label randomized controlled study. J. Assoc. Phys. India.

[31] Ferrarini A., Vacca A., Solimando A.G., Tavio M., Acquaviva R., Rocchi M., Nitti C., Salvi A., Menditto V., Luchetti Gentiloni M.M., Russo A., Moretti M., Pavani M., Giacometti A., Bonifazi M., Zuccatosta L., Romani L., Racanelli V., Moroncini G., Gabrielli A., Pomponio G. (2023). Early administration of tofacitinib in COVID-19 pneumonitis: an open randomised controlled trial. Eur. J. Clin. Invest..

[32] Almasi S., Rashidi A., Kachuee M.A., Shirazi B.M., Izadi S., Ghaffarpour S., Azimi M., Naghizadeh M.M., Makiani M.J., Ranjbar M., Goudarzi M., Rahimian N., Ghazanfari T. (2023). Effect of tofacitinib on clinical and laboratory findings in severe and resistant patients with COVID-19. Int. Immunopharm..

[33] Guimarães P.O., Quirk D., Furtado R.H., Maia L.N., Saraiva J.F., Antunes M.O., Kalil Filho R., Junior V.M., Soeiro A.M., Tognon A.P., Veiga V.C., Martins P.A., Moia D.D.F., Sampaio B.S., Assis S.R.L., Soares R.V.P., Piano L.P.A., Castilho K., Momesso R.G.R.A.P., Monfardini F., Guimarães H.P., Ponce de Leon D., Dulcine M., Pinheiro M.R.T., Gunay L.M., Deuring J.J., Rizzo L.V., Koncz T., Berwanger O. (2021). Tofacitinib in patients hospitalized with covid-19 pneumonia. N. Engl. J. Med..

[34] Sims J.T., Krishnan V., Chang C.Y., Engle S.M., Casalini G., Rodgers G.H., Bivi N., Nickoloff B.J., Konrad R.J., de Bono S., Higgs R.E., Benschop R.J., Ottaviani S., Cardoso A., Nirula A., Corbellino M., Stebbing J. (2021). Characterization of the cytokine storm reflects hyperinflammatory endothelial dysfunction in COVID-19. J. Allergy Clin. Immunol..

[35] Barlow-Pay F., Htut T.W., Khezrian M., Myint P.K. (2021). Systematic review of immunosuppressant guidelines in the COVID-19 pandemic. Ther Adv Drug Saf.

[36] Peng J., Fu M., Mei H., Zheng H., Liang G., She X., Wang Q., Liu W. (2022). Efficacy and secondary infection risk of tocilizumab, sarilumab and anakinra in COVID-19 patients: a systematic review and meta-analysis. Rev. Med. Virol..

[37] Seif F., Aazami H., Khoshmirsafa M., Kamali M., Mohsenzadegan M., Pornour M., Mansouri D. (2020). JAK inhibition as a new treatment strategy for patients with COVID-19. Int. Arch. Allergy Immunol..

[38] Liu Q., Zhou Y.H., Yang Z.Q. (2016). The cytokine storm of severe influenza and development of immunomodulatory therapy. Cell. Mol. Immunol..

[39] Hoisnard L., Lebrun-Vignes B., Maury S., Mahevas M., El Karoui K., Roy L., Zarour A., Michel M., Cohen J.L., Amiot A., Claudepierre P., Wolkenstein P., Grimbert P., Sbidian E. (2022). Adverse events associated with JAK inhibitors in 126,815 reports from the WHO pharmacovigilance database. Sci. Rep..

[40] Huang F., chun Luo Z. (2019). Adverse drug events associated with 5mg versus 10mg Tofacitinib (Janus kinase inhibitor) twice daily for the treatment of autoimmune diseases: a systematic review and meta-analysis of randomized controlled trials. Clin. Rheumatol..

[41] Song Y.K., Song J., Kim K., Kwon J.W. (2022). Potential adverse events reported with the Janus kinase inhibitors approved for the treatment of rheumatoid arthritis using spontaneous reports and online patient reviews. Front. Pharmacol..

